# Preventive digital mental health interventions for children and young people: a review of the design and reporting of research

**DOI:** 10.1038/s41746-020-00339-7

**Published:** 2020-10-15

**Authors:** Aislinn D. Bergin, Elvira Perez Vallejos, E. Bethan Davies, David Daley, Tamsin Ford, Gordon Harold, Sarah Hetrick, Megan Kidner, Yunfei Long, Sally Merry, Richard Morriss, Kapil Sayal, Edmund Sonuga-Barke, Jo Robinson, John Torous, Chris Hollis

**Affiliations:** 1 NIHR MindTech MedTech Co-operative, Institute of Mental Health, School of Medicine, University of Nottingham, Nottingham, UK; 2 NIHR Nottingham Biomedical Research Centre, Mental Health and Technology Theme, University of Nottingham, Nottingham, UK; 3 Division of Psychiatry and Applied Psychology, University of Nottingham, Nottingham, UK; 4 Centre for ADHD and Neurodevelopmental Disorders Across the Lifespan, Institute of Mental Health, School of Medicine, University of Nottingham, Nottingham, UK; 5 Department of Psychiatry, University of Cambridge, Cambridge, UK; 6 Faculty of Education, University of Cambridge, Cambridge, UK; 7 MRC Centre for Neuropsychiatric Genetics and Genomics, Division of Psychological Medicine and Clinical Neurosciences, Cardiff University, Cardiff, UK; 8 School of Psychology, Trinity College Dublin, Dublin, Ireland; 9 Department of Psychological Medicine, The University of Auckland, Auckland, New Zealand; 10 Orygen, University of Melbourne, Parkville, Australia; 11 The Children and Young People’s Mental Health Research Collaboration, University of Exeter, Exeter, UK; 12 Horizon Digital Economy Research Institute, University of Nottingham, Nottingham, UK; 13 School of Computer Science and Electronic Engineering, University of Essex, Colchester, UK; 14 NIHR Applied Research Centre East Midlands, Nottingham, UK; 15 Department of Child & Adolescent Psychiatry, Institute of Psychiatry, Psychology & Neuroscience, King’s College London, London, UK; 16 Department of Child & Adolescent Psychiatry, Aarhus University, Aarhus, Denmark; 17 Centre for Youth Mental Health, University of Melbourne, Parkville, Australia; 18 Department of Psychiatry, Beth Israel Deaconess Medical Center, Harvard Medical School, Boston, MA USA

**Keywords:** Psychology, Medical research

## Abstract

Digital health interventions (DHIs) have frequently been highlighted as one way to respond to increasing levels of mental health problems in children and young people. Whilst many are developed to address existing mental health problems, there is also potential for DHIs to address prevention and early intervention. However, there are currently limitations in the design and reporting of the development, evaluation and implementation of preventive DHIs that can limit their adoption into real-world practice. This scoping review aimed to examine existing evidence-based DHI interventions and review how well the research literature described factors that researchers need to include in their study designs and reports to support real-world implementation. A search was conducted for relevant publications published from 2013 onwards. Twenty-one different interventions were identified from 30 publications, which took a universal (*n* = 12), selective (*n* = 3) and indicative (*n* = 15) approach to preventing poor mental health. Most interventions targeted adolescents, with only two studies including children aged ≤10 years. There was limited reporting of user co-design involvement in intervention development. Barriers and facilitators to implementation varied across the delivery settings, and only a minority reported financial costs involved in delivering the intervention. This review found that while there are continued attempts to design and evaluate DHIs for children and young people, there are several points of concern. More research is needed with younger children and those from poorer and underserved backgrounds. Co-design processes with children and young people should be recognised and reported as a necessary component within DHI research as they are an important factor in the design and development of interventions, and underpin successful adoption and implementation. Reporting the type and level of human support provided as part of the intervention is also important in enabling the sustained use and implementation of DHIs.

## Introduction

In the UK, 25% of 17–19 year-olds experience significant levels of mental illness often accompanied by self-harm and sometimes escalating to suicide^1^. As 50% of mental health problems are established by the age of 14 and 75% by the age of 24^2^, prevention and early intervention in children and young people are of critical importance^3^. Preventive interventions include those that support children and young people to develop skills to maintain mental health, that target pre-clinical risk factors, or respond to early signs of distress^4^. This review will apply the Gordon framework of prevention to mental health problems: universal (targeting whole populations, regardless of current mental health status), selective (targeting specified risk factors) and indicated levels (targeting early sub-clinical signs and symptoms)^5^. Tertiary prevention, targeting existing mental health disorders, is not within the scope of this review.

The rapid growth of digital technologies (e.g. smartphones, wearables) has created the potential for predictive prevention: the use of data to personalise preventive interventions^6^. The ubiquity of digital technologies offers an opportunity to support increased access to mental health interventions for children and young people^7^. To date, there is little evidence to demonstrate the successful implementation and subsequent impact of evidence-based digital mental health interventions for children and young people at scale^8^. This scoping review aims to further our understanding of the challenges to implementation faced by digital health interventions (DHIs) addressing the prevention and early intervention of mental health problems in children and young people by examining the reporting of factors that improve opportunities for successful adoption into real-world contexts. These include features related to the development, evaluation and implementation of DHIs^9,10^.

Previous studies have identified several challenges to the use of DHIs in routine service delivery including technical difficulties and low awareness of data standards and privacy^11^, as well as low engagement and retention rates amongst users^12^. Moreover, several gaps have been identified in research such as the lack of economic evaluations and implementation studies^11,13^. A scoping review of mental health apps for young people documented several advantages of apps including ubiquity, flexibility, and timely communication, but these were challenged by technical difficulties, poor adherence, and few studies that addressed privacy or conducted an economic evaluation^14^. The large numbers of publicly available digital mental health interventions (over 10,000^8^) highlight the growing gap between research and evaluation as well as practice and implementation. For instance, a recent review found only 2 of 15 evidence-based mental health apps were available to download despite their acceptability^15^ and the clear need for effective DHIs in routine care^16^.

Efficacious DHIs for mental health have been reviewed across the age-span of children and young people^7^, including university students^17,18^, and in relation to those with anxiety disorders^19^ and/or depression^17,19–24^. However, generalising efficacy of DHIs outside research settings is constrained if interventions are not sufficiently appropriate or appealing, challenging not only the engagement of children and young people^25^ but potentially also those who support them (e.g. parents and teachers). Engaging stakeholders in the development, implementation and evaluation of technologies is one of the pillars for responsible research and innovation (RRI) and a crucial element to develop new digital innovations in a socially desirable and acceptable way^26^. For example, researchers must consider the wider societal implications (e.g., workforce issues, training/skills) as well as consider how real-world uptake might differ from usage within a trial (e.g., limited mobile data) as engagement is a key barrier to the effectiveness of DHIs in mental healthcare^8^. Co-production with young people, and where relevant their parents^25^, as well as specialist technical and psychological input^20^, is important in identifying and potentially mitigating these problems.

There is clearly potential for DHIs to be effective in prevention and early intervention for children and young people but there is little research addressing the opportunities and challenges of their adoption into real-world contexts. This review examines factors related to successfully sustained DHIs^9,10^ and applies these to evidence-based interventions designed to prevent mental health disorders in children and young people. These include mapping the characteristics of studies, their participants, design elements, and features related to implementation. It aims to identify potential influences of successful adoption within the existing evidence-base and highlights factors that should be addressed by researchers in the design and reporting of the development, evaluation and implementation of DHIs. A scoping review approach was chosen as it is more suitable than a systematic review where the purpose of the review is to identify knowledge gaps, scope a body of literature to investigate the adequacy of research design and reporting^27^.

## Methods and analysis

This scoping review uses the framework proposed by Arksey and O’Malley^28^ and is informed by PRISMA guidelines^29^.

### Search strategy

Five electronic databases (ACMDL, PubMed, PsycInfo, Embase and Cochrane Library) were searched in June 2019 for all full-text publications in English published from 2013 to 19/06/2019. Reviews were searched when identified and any relevant articles included. A previous systematic review by Clarke et al.^30^ reported on 28 studies conducted between 2000 and early 2013. These studies were followed up so as to map whether these interventions had been subject to further research.

### Study screening

Eligibility criteria were as follows:

- peer-reviewed studies
- DHIs for mental health disorders
- children and young people aged 0–25 years old
- universal, selective or indicated prevention
- participants were not included in studies on the basis of a clinical diagnosis (e.g. depression or anxiety)
- DHI delivered in any location

Screening was conducted by four researchers (AB, EBD, EPV and MK).

### Data Extraction

The data extraction chart was developed iteratively using the Joanna Briggs Institute template^31^ by three researchers (AB, EPV and EBD), with input from other authors, to reflect categories identified within the previous review^30^ and the literature surrounding digital therapies and prevention for children and young people. Thirty-four categories were identified ranging from demographic information to key elements of prevention, implementation and involvement. Three researchers co-ordinated to ensure that the coding framework was appropriate and consistently applied. Four main categories were identified – study characteristics, user characteristics, usability and engagement, and implementation – from the data extraction chart.

## Results

### Study selection

**Fig. 1:**
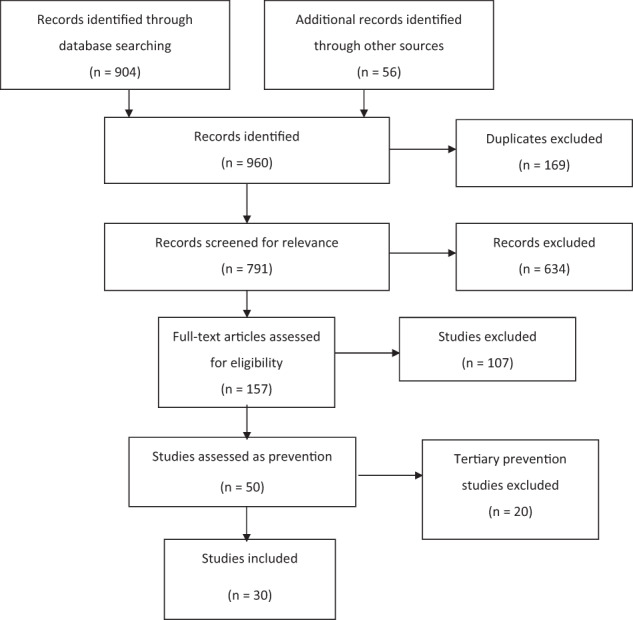
PRISMA flow diagram outlining the search process and the exclusion of ineligible articles.

Thirty studies were identified from 791 studies (Fig. 1).

### Study characteristics

The 30 studies included relate to 21 different interventions. These include 12 providing universal prevention (Table 1), 3 selective (Table 2) and 15 indicated prevention (Table 3). The majority of studies reported on interventions accessed via the internet (*n* = 21) while a small number used instant messaging (*n* = 2) or social media elements (*n* = 1). Some used an app (universal = 2, selective = 1), whilst those available only on a desktop or laptop computer (*n* = 8) mainly used game elements (*n* = 5).

**Table 1:** Study characteristics for universal prevention studies.

Citation	Country	Study Design	Comparator(s)	Population	Age range	Type of Intervention	Setting	Condition
Perry et al.^43^	Australia	RCT^a^	lifeSTYLE (offline intervention)	Students	15–18 years^b^	CBT^c^ and psychoeducation	School	General wellbeing
Kuosmanen et al.^44^	Australia	Qualitative	n/a^d^	Students	15–20 years	CBT and psychoeducation	Youth Centre	General wellbeing
Calear et al .^45^	Australia	Pilot RCT	WL^e^	Students	13–17 years	CBT and psychoeducation	Home	Anxiety
Calear et al.^46^	Australia	RCT	Supported by teachers or externally, unsupported, WL	Students	12–18 years	CBT and psychoeducation	School	Depression and anxiety
Burckhardt et al.^47^	Australia	Pilot RCT	Entertainment website	Students	12–18 years	Positive psychology, psychoeducation, mindfulness	School	General wellbeing
Bannink et al.^48^	Netherlands	RCT	Intervention only, intervention with consultation group, WL	Students	15–16 years	Psychoeducation and motivational interview	Home	General wellbeing
Bidargaddi et al.^56^	Australia	RCT	WL	General population	16–25 years	Not reported	Home	General wellbeing
Bidargaddi et al.^56^	Australia	RCT	WL	General population	16–25 years	Not reported	Home	General wellbeing
Lillevoll et al.^42^	Norway	Pilot	Tailored email, generic email, no email, WL	Students	15–25 years	CBT and psychoeducation	Home	Depression
Taylor-Rodgers et al.^57^	Canada	RCT	Online information	General population	18–25 years	Psychoeducation	Home	General wellbeing
Levin et al.^58^	USA	RCT	WL	Students	18–20 years	Acceptance commitment therapy	Home	General wellbeing
Rodriguez et al.^59^	Spain	Pilot	n/a	Adolescents	9–14 years	Emotional regulation	Lab	General wellbeing
Whittaker et al.^41^	Australia	RCT	Control version of intervention	General population	13–17 years	CBT	Home	General wellbeing

^a^Randomised controlled trial.

^b^Years of age.

^c^Cognitive behavioural therapy.

^d^Not applicable.

^e^Waitlist.

**Table 2:** Study characteristics for selective prevention studies.

Citation	Country	Study Design	Comparator(s)	Population	Age range	Type of intervention	Setting	Condition
Woolderink et al.^49^	Netherlands	Qualitative	n/a^a^	Children of parents with addictions or a mental health disorder	16–25 years^b^	Online therapy	Home	General wellbeing
Boring et al.^50^	USA	RCT^c^	Best of the Net self-study	Children of divorced parents	11–17 years	CBT^d^ and psychoeducation	Home	General wellbeing
Lattie et al.^32^	USA	Pilot	n/a	Adolescents at risk of depression or substance misuse	14–19 years	CBT	Home	Depression

^a^Not applicable.

^b^Years of age.

^c^Randomised controlled trial.

^d^Cognitive behavioural therapy.

**Table 3:** Study characteristics for indicated prevention studies.

Citation	Country	Study Design	Comparator(s)	Population	Age range	Type of intervention	Setting	Condition
Sethi^51^	Australia	RCT^a^	Face-to-face CBT^b^, intervention only, combination	Young adults with mild to moderate depression and/or anxiety	18–25 years^c^	CBT and psychoeducation	Multiple	Depression and anxiety
Robinson et al.^52^	Australia	Pilot	n/a^d^	Students at risk of suicide	14–18 years	CBT and psychoeducation	Multiple	Suicidal ideation
Robinson et al.^53^	Australia	Pilot	n/a	Students at risk of suicide	14–18 years	CBT and psychoeducation	Multiple	Suicidal ideation
Hetrick et al.^54^	Australia	RCT	Treatment as usual	Students at risk of suicide	15–19 years	CBT and psychoeducation	Multiple	Suicidal ideation, depression and anxiety
Stasiak et al.^55^	New Zealand	Pilot RCT	Computerised attention placebo and psychoeducation	Adolescents with mild to moderate depression	13–18 years	CBT and psychoeducation	School	Depression
Poppelaars et al.^60^	Netherlands	RCT	Op Volle Kracht (offline intervention), SPARX, OVK + SPARX and a monitoring control	Adolescents with elevated depression symptoms	11–16 years	CBT	Home	Depression
Lucassen et al.^61^	New Zealand	Pilot	n/a	Sexual minority youth with elevated depression symptoms	13–19 years	CBT	Multiple	Depression
Smith et al.^62^	UK	RCT	Wl	Adolescents with elevated depression symptoms	12–15 years	CBT	School	Depression
March et al.^63^	Australia	Pilot	n/a	Adolescents with elevated anxiety symptoms	7–17 years	CBT	Home	Anxiety
Eisen et al.^64^	USA	Pilot	n/a	Adolescents with elevated depression symptoms	14–21 years	CBT and interpersonal psychotherapy	Home	Depression
Gladstone et al.^65^	USA	RCT	Motivational interview and brief advice	Adolescents with elevated depression symptoms	14–21 years	CBT and interpersonal psychotherapy	Multiple	Depression
Ip et al.^66^	Hong Kong	RCT	Anti-smoking website	Adolescents with elevated depression symptoms	13–17 years	CBT and interpersonal psychotherapy	Home	Depression
Kramer et al.^67^	Netherlands	RCT	Wl	Adolescents with elevated depression symptoms	12–22 years	Solution-focused based therapy	Home	Depression
Rickhi et al.^68^	USA	Pilot RCT	Control	Adolescents with elevated depression symptoms	14–22 years	Spirituality	Home	Depression
Sportel et al.^69^	Netherlands	RCT	School delivered CBT, internet CBM^e^, and control	Adolescents with elevated anxiety symptoms	12–15 years	Cognitive bias modification	Home	Anxiety

^a^Randomised controlled trial.

^b^Cognitive behavioural therapy.

^c^Years of age.

^d^Not available.

^e^Cognitive bias modification.

The majority of participants were recruited from secondary education (*n* = 19; universal = 9, selective = 1, and indicated = 9). This was followed by primary healthcare (*n* = 4; selective = 1, and indicated = 3), universities (*n* = 3; universal = 2, and indicated = 1) and via the media (*n* = 3; universal = 1, and indicated = 2). One selective study identified potential participants through court documents.

### User characteristics

The majority of studies did not report participant characteristics that are known risk-factors for mental health disorders (*n* = 16). These included living status, education of participants and/or parents, technology ownership, and rural-urban classification (Tables 4–6). Selective studies identified those with parents who were divorced, or had addictions or mental illness, and those at risk of depression. Just under half of the studies did not report information about participants’ ethnicity (*n* = 15). Those that did were inconsistent in how this was measured, with seven simply identifying ‘local’ and ‘non-local’ participants.

**Table 4:** Individual characteristics, usability and engagement, and implementation features within reported universal prevention studies.

Citation	Intervention name	Ethnicity	SES	Other risk factors	Data ethics	User experience	User involvement	Implementation
Perry et al.^43^	SPARX-R		✓					
Kuosmanen et al.^44^	*SPARX-R (2)*					✓		✓
Calear et al.^45^	e-couch anxiety and worry		✓			✓		✓
Calear et al.^46^	e-couch anxiety and worry (1)		✓					
Burckhardt et al.^47^	Bite Back				✓			✓
Bannink et al.^48^	E-health4Uth							✓
Bidargaddi et al.^56^	The Toolbox					✓	✓	✓
Lillevoll et al .^42^	MoodGYM				✓			
Taylor-Rodgers et al.^57^	Psychoeducation for Help Seeking	✓				✓		✓
Levin et al.^58^	Acceptance and Commitment Therapy	✓					✓	✓
Rodriguez et al.^59^	GameTeen System				✓			
Whittaker et al.^41^	MEMO CBT

**Table 5:** Individual characteristics, usability and engagement, and implementation features reported within selective prevention studies.

Citation	Intervention Name	Ethnicity	SES	Other risk factors	Data ethics	User experience	User involvement	Implementation
Woolderink et al.^49^	Kopstoring			✓		✓		✓
Boring et al.^50^	Children of Divorce Coping with Divorce			✓		✓		✓
Lattie et al.^32^	ProjectTECH			✓		✓	✓	✓

**Table 6:** Individual characteristics, usability and engagement, and implementation features reported within indicated prevention studies.

Citation	Intervention name	Ethnicity	SES	Other risk factors	Data ethics	User experience	User involvement	Implementation
Sethi^51^	MoodGYM							✓
Robinson et al.^52^	Reframe-IT		✓					✓
Robinson et al.^53^	Reframe-IT (2)					✓		
Hetrick et al.^54^	Reframe-IT (3)							
Stasiak et al.^55^	The Journey					✓		✓
Poppelaars et al.^60^	SPARX		✓			✓		✓
Lucassen et al.^61^	Rainbow SPARX	✓	✓	✓		✓	✓	✓
Smith et al.^62^	Stressbusters							✓
March et al.^63^	BRAVE Self Help		✓			✓		
Eisen et al.^64^	CATCH-IT	✓	✓					
Gladstone et al.^65^	CATCH-IT (2)	✓	✓		✓			
Ip et al.^66^	Grasp the Opportunity (3^a^)							
Kramer et al.^67^	PratenOnline							✓
Rickhi et al.^68^	LEAP						✓	
Sportel et al.^69^	Internet delivered Cognitive Bias Modification							

^a^Grasp the Opportunity is a Chinese-language version of CATCH-IT.

Universal interventions were aimed at young people whose ages ranged from 9 to 25 years old (mean = 17.35, SD = 3.97) whilst selective interventions were for between 11 and 25 years old (mean = 17.47, SD = 3.75). Indicated interventions included the broadest age range from 7 to 25 years old (mean = 16.15, SD = 3.27). The majority of interventions targeted participants between the ages of 15–16 years (*n* = 22, mean = 16.73, SD = 3.59). No interventions were aimed at children aged ≤6 years. Universal and selective primary prevention interventions were also not available to children aged ≤9 years.

### Usability and engagement

Most studies reported capturing user experience through questionnaires (*n* = 9) that range from a series of questions around helpfulness and acceptability, to a single question asking if the user is satisfied. Only one study reported using multiple validated measures—the System Usability Scale and the Usefulness, Satisfaction and Ease of use (USE) questionnaire^32^. Other methods used included interviews (*n* = 3), focus groups (*n* = 2) and surveys (*n* = 1). Only six interventions reported on user involvement in the development of interventions. This included collaborative working on the content and design of the intervention with young people and other users, involvement in usability testing, and engagement of young people with lived experience to feedback on materials.

Only 8 studies identified completion criteria. Some used automated methods, such as the number of logins, to assess engagement (*n* = 7). Others used self-report (*n* = 2) or relied on attendance (e.g. interventions delivered within schools). Because most interventions did not report on how much of an intervention needed to be completed, it was difficult to identify whether participants have been sufficiently engaged. Reasons provided for dropout are linked to setting (e.g. school absence) and technical issues (e.g. logging in difficulties). Other factors included older age, higher and lower levels of anxiety, speaking a language other than English, feeling better or too unwell, being busy, or responding badly to the intervention.

### Implementation

Most universal studies (*n* = 11/12) recruited all those that chose to participate who were within the age range. Only one study screened participants using a clinical interview for depression within the general school population, for the purpose of excluding those in the clinical range and identifying changes within a clinical score. Universal studies were delivered at home (*n* = 7), within schools (*n* = 4), and a lab (*n* = 1). Selective studies targeted specific at-risk populations (e.g. children of divorced parents) and were all delivered within a home setting (*n* = 3). Six of the fifteen indicated studies used clinical assessments to identify participants and these interventions were delivered in school (*n* = 1), multiple settings (*n* = 3) and home (*n* = 2). Nine used self-reported assessments and were delivered within the home (*n* = 5), multiple settings (*n* = 3) and schools (*n* = 1).

All indicated (*n* = 15) and one selective study included participants with a score indicating mental health symptomology. Studies variously excluded those with a mental health diagnosis, low levels of comprehension, those currently in treatment, those with scores that were too high or low on mental health symptomology, those not exclusively attracted to the opposite sex, and those with current suicidality.

The barriers and facilitators to implementation were different for those programmes delivered within schools, at home and across multiple settings. Within schools, completion was often challenged by absences. Relevance was a key issue across all settings with involved stakeholders needing to understand the programme and its outcomes, and feel that it was relevant to their experience. It was also necessary for the preventive DHI to be provided through an easily accessible and appropriate device. For instance, within schools it may be more difficult to implement app-based interventions due to restrictions on students’ smartphone use or it may create inequalities for those students without access to one. Technical issues across all settings challenged use; this was overcome when support was provided by researchers to those delivering (e.g. teachers) the intervention. Much of the data focused on implementation was around the acceptability, usability and content of the programme, rather than challenges and opportunities in real-world dissemination. Consent was challenging in two studies where parental consent was needed for those disclosing their sexuality, and another, delivered remotely, that noted some participants did not realise they were engaged in a research study. Despite the potential for DHIs to be delivered without clinical oversight only a small number of studies (*n* = 3) reported adverse events or potential negative impact in detail.

The research was funded by fellowships, granted to researchers, and national health research grants. Funding was also received from accelerator programmes, charitable organisations, and was mainly from research, private and health funding with a small amount of business funding. Some funding was received for developing the intervention, and others received funding for delivery of the research, but it was mainly unclear what this funding was provided for. Although the reports include statements that there were no conflicts of interest, the founders and developers of interventions were at times involved in the research.

Only six studies reported the cost of their intervention within the study. The current availability of interventions was difficult to assess as many were difficult to search because they had names associated with other websites (e.g. ‘stressbusters’) or were not named within the research. Only 38% (*n* = 8/21) are publicly available. Of those accessible online some were only available in certain countries (e.g. SPARX is currently not available in the United Kingdom) or specific languages (e.g. Kopstoring is only available in Dutch). Intervention costs were at times reported online (e.g. MoodGYM) but it was difficult to ascertain how to access these interventions as an individual because they were often country-specific or required a significant amount of personal information (e.g. e-Couch).

## Discussion

DHIs offer the opportunity to provide children and young people with evidence-based interventions that prevent mental health disorders at an early stage. However, our scoping review suggests that DHIs are not yet meeting their potential and the design and reporting of research does not generally support real-world implementation. Whilst they are being delivered within several settings, studies most frequently recruited from secondary schools, colleges or universities. Younger children and those who do not engage in school or are often absent are not being included within research designs. Other known mental health risk-factors are not reported such as socio-economic status and ethnicity, and this limits the generalisability of the findings to populations where there are most need and potential benefit. This is particularly significant as ethnic minorities are less likely to be referred to or access formal mental health services^33–35^. Reporting participants’ demographic characteristics are essential to know if research is engaging with groups hardly reached (e.g., sexual and gender minorities) which often are at increased risk for mental health problems, including suicide^36^. Demographic information can also signal if equality, diversity and inclusion strategies have been applied to understand how results may generalize to different groups; thereby supporting adoption and implementation.

DHIs are designed mainly to deliver cognitive behavioural therapy (CBT) for indicated prevention to target depression and/or anxiety symptoms. This is unsurprising considering CBT is the recommended treatment for depression^37^ and anxiety^38^ in children and young people and the most studied intervention offline for anxiety and depression^39^. Fewer interventions aimed to improve general wellbeing, which suggests researchers may be more likely to develop interventions based on existing clinical guidelines despite the effectiveness of tackling general wellbeing in non-digital prevention interventions^24^.

Preventive DHIs can be flexible in how they are offered; whether in terms of when, where or how they are accessed. However, different settings may offer unique challenges and opportunities that prevent individuals or those delivering from benefiting. Our review found that few studies reported on factors related to implementation and this represents an important gap in understanding how DHIs are best adopted to support the prevention of mental health disorders in children and young people. Our findings suggest there are two key areas that must be addressed: systemic understanding of how technical issues can be solved/supported and ensuring that the programme content is not only relevant to children and young people but also to those supporting its use (e.g. parents and teachers). It is also important to consider the real-world contexts into which these DHIs might be implemented. For instance, when delivered remotely it is not possible to apply consistent eligibility criteria and yet only three studies address adverse events or the negative impact of the DHI. Co-production of content, design and usability can increase the likelihood of successful implementation within DHIs and RRI frameworks recommend user involvement in the design of new technologies. However, within this review, only 10 interventions reported on user experience and five interventions reported that users had been involved in the research. It is clear that there is a significant lack of consensus as to how the user experience should be captured or involvement reported.

Regarding sustainability, there is a need to design, develop and test interventions within an implementation framework; i.e. with the pipeline of adoption firmly sitting as the foundation of the work. Real-world accessibility of interventions was difficult to ascertain as many are only available in specific countries or languages and the costs of access are not always clear. While these DHIs hold great potential to be disseminated and used widely, our review indicates that too often software is not updated, and existing interventions do not take advantage of newer developments that have the potential to improve the predictive capacity of preventive interventions, such as sensors and wearables, machine learning or natural language processing methodologies^40^.

Another issue highlighted is that programme completion and reasons for dropout need to be addressed more clearly as it is difficult to assess how many modules or how engaged a user needs to be for it to be considered complete. Outcomes are potentially impacted by the type of technology used and the way in which data are collected. Many DHIs still rely on self-report, which is less objective and considered scientifically less rigorous, despite the potential for automated measurements. One study demonstrated high rates of follow up (MEMO—98% post-intervention and 92.5% at 12 months) that were attributed to strategies they developed to reduce dropout^41^. Another (MoodGYM, delivered nationally) had extremely low rates of engagement (8.5% logged into the first module)^42^. However, this may be due to differences in how this was measured and the method of delivery. The latter was done so automatically through logging access to the website whilst the former relied on self-report. Whether either can accurately capture engagement remains to be seen.

### Strengths and limitations

This scoping review examined 30 studies representing 21 different DHIs for the prevention of mental health disorders amongst children and young people delivered in various settings. Our results provide an updated summary of factors related to the adoption of interventions into real-world contexts, reporting on stakeholder involvement, engagement and users’ experience. We have also expanded on past reviews by identifying the potential for these prevention interventions to be disseminated and used widely. This review has identified tools currently used to address user experience and engagement within these interventions and highlighted the gaps in the design and reporting of research (e.g., reporting risk factors, gaps in age range covered by interventions). We have mapped barriers and facilitators to real-world implementation including differences between research and practice (e.g., exclusion criteria, implementation, funding and costs). The findings of this review highlight that there is more work needed to address better research design and reporting of development, evaluation and implementation of DHIs for the prevention of mental health disorders in children and young people if their potential is to be fully realised.

The strength of this scoping review is that it looked broadly at mental health prevention across multiple settings, including user experience and involvement, implementation or the real-world uptake of interventions. However, it did not include grey literature and there may have been co-design and involvement within studies that went unreported in publications. An agreed DHI taxonomy would be beneficial to identify common core components between interventions. Their clinical, technical and evaluative mechanisms are reported in several ways, which challenges this reviews clarity.

### Recommendations

More research is needed examining factors related to the successful adoption of preventive DHIs for children and young people within mental health and how they can be encouraged; addressing risk factors, ethical issues including consent processes for remote delivery, and younger age groups. We recommend that researchers report the amount of their programme that must be completed (minimum dose) and identify the availability and accessibility metrics of their intervention including costs (e.g. through an economic evaluation). Real-world implementation is imperative to consider^8^, and more research should address this within different settings and technologies. DHI research would also benefit from an agreed taxonomy for reporting the clinical, technical and evaluative components. The impact of these interventions, including negative reactions or the exclusion of certain populations, must also be addressed in future research. Finally, we recommend that funding is provided that ensures the sustainability of research-based DHIs from development through to real-world dissemination.

## Supplementary information

Supplementary Information

## Data Availability

The data that support the findings of this study are available from the corresponding author upon reasonable request.
