# Supplementary material for: Preventive digital mental health interventions for children and young people: a review of the design and reporting of research
Source: NPJ Digit Med. 2020 Oct 15;3:133. doi: 10.1038/s41746-020-00339-7 (PMC7562906; doi:10.1038/s41746-020-00339-7)
Supplement: Supplementary file 1 — Supplementary Information [file 41746_2020_339_MOESM1_ESM.pdf]

	**Search terms**
**Prevention**	Prevent? OR promot?
**Technology**	“E-mental health” OR “Digital Mental Health” OR “Digital Health Technologies” OR Internet OR “eHealth” OR mobile OR computer? OR smartphone OR “social media” OR online OR virtual OR "mhealth" OR electronic
**Population**	“Young adult?” OR Kid? OR Child? OR Teen? OR Pupil OR Adolescen? OR “Young people” OR youth OR school OR parent? OR family
**Intervention**	Intervention OR Treatment OR Therapy OR Training OR programme OR review
**Mental Health**	"mental health" OR “Mental distress” OR “Mental illness” OR “Mental difficulties” OR “Mental issues” OR Depressi? OR Anx? OR “Eating disorder” OR Self-harm OR Suicid? OR “suicidal ideation” OR “Neurodevelopmental disorders” OR Psychosis OR ADHD OR Autism OR ASD OR “Language delay” OR “Conduct problems” OR “Peer problems” Or “School problems”
